# Effect of exercise‐induced muscle damage on vascular function and skeletal muscle microvascular deoxygenation

**DOI:** 10.14814/phy2.13032

**Published:** 2016-11-24

**Authors:** Jacob T. Caldwell, Garrett C. Wardlow, Patrece A. Branch, Macarena Ramos, Christopher D. Black, Carl J. Ade

**Affiliations:** ^1^ Department of KinesiologyKansas State UniversityManhattanKansas; ^2^ Department of Health and Exercise Sciencethe University of OklahomaNormanOklahoma

**Keywords:** Flow‐mediated dilation, muscle damage, near‐infrared spectroscopy, vascular function

## Abstract

This paper investigated the effects of unaccustomed eccentric exercise‐induced muscle damage (EIMD) on macro‐ and microvascular function. We tested the hypotheses that resting local and systemic endothelial‐dependent flow‐mediated dilation (FMD) and microvascular reactivity would decrease, $\dot{V}O_{2\max}$ would be altered, and that during ramp exercise, peripheral O_2_ extraction, evaluated via near‐infrared‐derived spectroscopy (NIRS) derived deoxygenated hemoglobin + myoglobin ([HHb]), would be distorted following EIMD. In 13 participants, measurements were performed prior to (Pre) and 48 h after a bout of knee extensor eccentric exercise designed to elicit localized muscle damage (Post). Flow‐mediated dilation and postocclusive reactive hyperemic responses measured in the superficial femoral artery served as a measurement of local vascular function relative to the damaged tissue, while the brachial artery served as an index of nonlocal, systemic, vascular function. During ramp‐incremental exercise on a cycle ergometer, [HHb] and tissue saturation (TSI%) in the *m. vastus lateralis* were measured. Superficial femoral artery FMD significantly decreased following EIMD (pre 6.75 ± 3.89%; post 4.01 ± 2.90%; *P* < 0.05), while brachial artery FMD showed no change. The [HHb] and TSI% amplitudes were not different following EIMD ([HHb]: pre, 16.9 ± 4.7; post 17.7 ± 4.9; TSI%: pre, 71.0 ± 19.7; post 71.0 ± 19.7; all *P* > 0.05). At each progressive increase in workload (i.e., 0–100% peak), the [HHb] and TOI% responses were similar pre‐ and 48 h post‐EIMD (*P* > 0.05). Additionally, $\dot{V}O_{2\max}$ was similar at pre‐ (3.0 ± 0.67 L min^−1^) to 48 h post (2.96 ± 0.60 L min^−1^)‐EIMD (*P* > 0.05). Results suggest that moderate eccentric muscle damage leads to impaired local, but not systemic, macrovascular dysfunction.

## Introduction

Periods of intense and prolonged unaccustomed eccentric muscle activity place excessive strain on the myofibrils, altering their structure and function (Clarkson et al. 1987; Clarkson and Hubal 2002; Davies et al. 2011a). This phenomenon is often referred to as exercise‐induced muscle damage (EIMD) and results in a decreased force production, loss in range‐of‐motion, edema, and delayed‐onset soreness in the exercised muscles (Malm 2001; Hubal et al. 2008; Burt and Twist 2011; Davies et al. 2011a; Burt et al. 2013; Black et al. 2015). In addition to the acute decline in skeletal muscle function, mounting evidence demonstrates that EIMD also impacts performance during endurance exercise. Both time‐trial (Marcora and Bosio 2007; Twist and Eston 2009; Burt and Twist 2011) and time‐to‐exhaustion (Asp et al. 1998; Carmichael et al. 2006, 2010; Davies et al. 2008, 2009, 2011b) performance decline following EIMD. However, the mechanism(s) by which EIMD reduces performance remain unresolved. Reduced movement economy (Burt et al. 2013, 2014), heightened ratings of perceived exertion (RPE) (Davies et al. 2009; Twist and Eston 2009; Black and Dobson 2012; Burt et al. 2013), acute (Black and Dobson 2012), and prolonged reductions (Black et al. 2015) in $\dot{V}O_{2\mathrm{peak}}$ have also been observed in the days following EIMD. However, the decreased $\dot{V}O_{2\mathrm{peak}}$ following EIMD is not universal (Warren et al. 1999; Davies et al. 2011b).

One area that has received less attention, especially in humans, as a potential mechanism of EIMD's impact on endurance performance is the impact of damage and localized inflammation on macro‐ and microvascular circulation. EIMD has been shown to elicit an acute increase in large central arterial stiffness that is associated with the magnitude of muscle damage (Barnes et al. 2010). Stacy et al. (2013) have also demonstrated that EIMD of the biceps brachii decreased brachial artery endothelial‐dependent and independent dilation at 48 h. This suggests that EIMD alters local large artery endothelial function, and when coupled with the previous reports of central artery stiffness implies that vascular dysfunction may exist both locally and systemically. In addition to macrovascular dysfunction, some studies also report significant capillary hemodynamic changes following EIMD. Kano et al. (2004) demonstrated in rats, following electrically stimulated eccentric contractions, that significant changes in capillary lumen shape and distensibility occur. These authors later report significant decreases in red blood cell flux and velocity in the days following EIMD elicited via downhill running (Kano et al. 2005). These finding suggest that a degree of microvascular dysfunction may also be present following EIMD. In humans, near‐infrared spectroscopy (NIRS) has been used to evaluate peripheral O_2_ extraction, via changes in deoxygenated hemoglobin+myoglobin ([HHb]) (Chin et al. 2011; Boone et al. 2016) and tissue oxygenation (i.e., O_2_ supply to O_2_ demand) (Spencer et al. 2012) during cycling exercise. Davies et al. (2008) demonstrated that EIMD slows the [HHb] onset kinetics and the oxygen delivery/utilization ratio ($\dot{Q}O_{2}$:$\dot{V}O_{2}$), but did not change $\dot{V}O_{2}$ kinetic responses 48 h following eccentric damage. These findings suggest EIMD may shift the $\dot{Q}O_{2}$:$\dot{V}O_{2}$ ratio in the early phases of exercise in an attempt to preserve blood–myocyte O_2_ flux in the face of an increasing $\dot{V}O_{2}$. However, unlike the exponential increase in [HHb] observed during the step‐transition to exercise steady state (i.e., NIRS kinetic response), the [HHb] response during ramp‐incremental exercise varies as a function of continuous dynamic adjustments to changes in workload (Ferreira et al. 2007; Spencer et al. 2012; Bellotti et al. 2013; Fontana et al. 2015; Keir et al. 2015). Thus, the NIRS response during incremental exercise would provide additional insight into microvascular function, based upon Δ[HHb] and ΔTOI%, beyond that previously observed following EIMD.

To date, no study has examined the effects of EIMD on local and systemic macrovascular function as well as microcirculatory function during ramp‐incremental exercise in the days following moderate eccentric muscle damage. Impairments in either macro‐ or microvascular function could lead to reductions in $\dot{V}O_{2\mathrm{peak}}$. Accordingly, the primary aim of this study was to determine whether eccentric exercise has an unfavorable effect on macro‐ and microvascular function. Based on previous findings, (1) we hypothesized that local and systemic endothelial‐dependent flow‐mediated dilation would be decreased; (2) the muscle activation, peripheral O_2_ extraction, and $\dot{V}O_{2}$ responses (using surface electromyography, NIRS, and gas exchange, respectively) during ramp‐incremental exercise would be altered 48 h following EIMD elicited via unaccustomed eccentric exercise.

## Materials and Methods

### Participants

Thirteen participants (men *n* = 10; women *n* = 3) (age 21 ± 4 years [mean ± SD]) completed the study. Participants were free from known cardiovascular, pulmonary, metabolic, bone or joint disease, and were nonsmokers as determined via health history questionnaire. All participants completed less than 5 h of physical activity per week, had undertaken no structured lower body resistance training or long distance endurance training for at least 6 months prior to the study. Written informed consent was obtained from all individuals included in the study according to the University of Oklahoma Institutional Review Board for Research Involving Human Subjects requirements. Participants were instructed to refrain from taking antiinflammatory medication, ingestion of caffeine, and all exercise during the course of the study.

### Experimental design

All subjects reported to the laboratory, after a 12‐h overnight fast, on three separate days, at the same time of day ±30 min to prevent diurnal variations in the measured responses. Women were tested in the early follicular phase of their menstrual cycle (Gaebelein and Senay 1982). All testing sessions were performed in a temperature‐controlled laboratory (21–22°C). On day 1, and day 3, pre‐ and 48 h after the eccentric muscle damaging exercise, respectively, brachial and superficial femoral artery endothelial‐dependent flow‐mediated dilation (FMD) were examined in random order under resting conditions followed by an incremental cycling exercise test to volitional exhaustion to determine maximal oxygen uptake ($\dot{V}O_{2\max}$). During the incremental test, muscle microvascular function, Δ[HHb], were assessed via near‐infrared spectroscopy.

On day 2, eccentric knee extension exercise was used to evoke muscle damage. Participants first performed three maximal isometric force trials in each leg, separated by ~2 min of rest. Participants then performed six sets of eight maximal eccentric contractions, with ~2 min of rest between sets, on a commercially available dynamometer (KIN‐COM, Chattanooga, TN). Participants were instructed to maximally resist the lever arm, which was moving at 20° per sec from 160° to 90° and lasted ~3 sec. Following eccentric lowering, the lever arm was passively returned for the succeeding contractions. After six sets, participants were given 2–3 min of recovery followed by a maximal isometric concentric contraction to observe if maximal isometric force had dropped ~50% relative to predamage measurements (see Assessment of muscle damage below). If subjects did not reach this threshold, another two eccentric sets were repeated until the maximal isometric strength was equal to or below 50% of the initial value.

### General procedures

#### Assessment of muscle damage

Pre‐ and 48 h post eccentric exercise, perceived muscle soreness/pain was assessed with the visual analog scale (VAS), which is a continuous 10‐cm scale anchored by two verbal descriptors labeled from the left (no pain) to the right (worst possible pain). While performing a squat to parallel (~90° knee angle), the participant was asked to place a line on the VAS at the point that represented their pain intensity (Sriwatanakul et al. 1982). Pain intensity was quantified by measuring the distance in centimeters from the “no pain” anchor and the subjects marked line. Following the VAS, a noninvasive objective evaluation of muscle damage, via a decrease in maximal isometric concentric force, was measured. Thus, participants performed three maximal isometric unilateral contraction trials for each limb in a seated upright position at a knee joint angle of 110°. Participants were given ~2–3 min between each trial to limit muscular fatigue. Isometric strength was recorded as the highest isometric force produced by each leg in both pre‐ and 48 h postconditions.

#### Endothelial‐dependent flow‐mediated dilation

Following a 15‐min supine rest period, brachial artery blood pressure was measured (Omron BP785, Omron healthcare Inc., Hoffman, IL). Subsequently, following reported guidelines, brachial and superficial femoral artery endothelial function were examined via flow‐mediated dilation in the right arm and leg, respectively (Harris et al. 2010). The order of testing was randomized with 10 min between tests. To assess the brachial artery, the right arm was abducted to an angle of ~80° from the torso at heart level and placed on a foam pad and insonated approximately midway between the antecubital and axillary regions. For the superficial femoral artery, the right leg was supported on a ~14 cm high platform with the superficial femoral artery insonated 4–5 cm distal to the bifurcation of the common femoral artery in accordance with previous investigations (Thijssen et al. 2006, 2008a,b). In both instances, a pneumatic rapid inflation/deflation cuff (D.E. Hokanson, Bellevue, Wash) was placed immediately distal to the probe. Measurements of artery diameter and blood velocity were simultaneously obtained with an ultrasound system equipped with a multi‐frequency linear array transducer operating at 10.00 MHz, placed ~10 cm from the antecubital fossa, with marks placed on both ends of the probe for subsequent measures 48 h post (Vivid 3; GE medical Systems, Milwaukee, WI). In all instances, the Doppler sample volume was set at the full width of the vessel and the insonation angle maintained <60°. Following a 1‐min baseline period, the pneumatic cuff was inflated to >250 mmHg for 5 min. Fifteen seconds prior to cuff inflation, blood velocity and diameter measurements began and continued through a 2‐min post occlusion phase. Brachial and superficial femoral diameters were measured using a commercially available edge‐detection and wall‐tracking software package (Vascular research tools 6, [Medical Imaging Applications, Coraville, Iowa]), while this software allows less intrareader variability, and reduces investigator bias, it is not without limitations (Williamson et al. 2008). Ultrasound data were collected at 15 frames per second and averaged into 3 sec bins for analysis. From initial baseline values, FMD peak absolute (mmΔ) and relative (%Δ) diameter changes were calculated post cuff release in the superficial femoral and brachial arteries. In all instances, the peak dilatory response was observed within 120 sec. Baseline and postcuff release time‐averaged mean velocity (cm sec^−1^) values per 3 sec were calculated using the manufacturers on‐screen software and time aligned with diameter to calculate shear rate [Shear rate (sec^−1^) = 4 × mean blood velocity (cm sec^−1^)/diameter (cm)]. Following cuff deflation, the stimulus for arterial dilation was calculated as the area under the shear rate curve to peak dilatory response (AUC_SR_) using the trapezoid rule as previously described (Harris et al. 2010). The postocclusive reactive hyperemic response was assessed as the peak hyperemic velocity of the first complete envelope obtained after cuff release and used to assess microvascular reactivity as previously described by Meredith et al. (1996).

#### $\dot{V}{\text{O}}_{2\max}$

Upon completion of the FMD tests, participants rested for ~15 min, then performed a ramp‐incremental exercise protocol on a cycle ergometer (Lode BV, Groningen, The Netherlands). The test began after participants were instructed to pedal at 60 revolutions per minute (rpm) with a 1‐min resting baseline followed by 2 min at 20 W, then a progressive increase in power output at a rate of 20 W min^−1^ until volitional exhaustion began. Metabolic and ventilatory variables were measured throughout the incremental cycling test (ParvoMedics Inc. Sandy, Utah) and averaged across 15 sec epochs. The system was calibrated prior to each test according to manufacturer's instructions. Following a 20‐min passive recovery, participants performed a constant‐power test to validate the attainment of $\dot{V}O_{2\max}$ during the initial incremental test. The work load was set to 20 W above the peak power output (PPO) achieved in the initial incremental test. Participants were instructed to cycle to exhaustion at 60–70 rpm, which in all cases was ≥2 min (Poole et al. 2008). The highest 15 sec mean average $\dot{V}O_{2}$ from the incremental test was considered a validated measurement of $\dot{V}O_{2\max}$ if it was within 200 mL × min^−1^ of the highest 15 sec mean average $\dot{V}O_{2}$ obtained during the constant‐power test (Poole et al. 2008).

#### Near‐Infrared spectrometry (NIRS)

Total hemoglobin + myoglobin concentration ([Hb]total) was computed, while oxygenated ([HbO_2_]) and deoxygenated ([HHb]) concentrations were measured in each individual using a rapid sampling frequency‐domain multi‐distance near‐infrared spectrometer system (Oxiplex TS, ISS, Champaign, IL). The NIRS device consisted of four infrared‐emitting diodes operating at wavelengths of 750 and 830 nm and one concurrent detector bundle (source‐distance = 2.5–4.0 cm). The NIRS probe was placed longitudinally on the belly of the right *m. vastus lateralis* and secured with a cohesive bandage. Accurate NIRS probe placement, to observe striation in vivo and depth of the muscle tissue, was determined using B‐mode 2D ultrasound. Prior to data collection and NIRS placement, the device was calibrated according to manufacturer's specifications. Throughout the test, NIRS data were captured at a frequency of 25 Hz and averaged into 1 sec bins during offline analysis.

The muscle microvascular dynamic [HHb] response to incremental exercise was taken as an estimate of skeletal muscle fractional O_2_ extraction as previously described by Ferreira et al. (2006, 2007); Spencer et al. (2012); Boone et al. (2016). Additionally, tissue saturation index (TSI%) was calculated as TOI = [HbO_2_]/([HbO_2_] + [HHb]) × 100 (Ferrari et al. 2011). The 1 sec values for [HHb] and TSI% were used to calculate a mean average across 30 sec bins centered upon 10% increments of the change in work rate from the 20 W (0%) to end exercise (100%). While previous investigations have used sigmoid and double linear models to evaluate changes in these NIRS responses in healthy men (Spencer et al. 2012), the present method, based upon work rate, has been used to obtain evaluation of the NIRS response across the complete range of metabolic rates, thus, allowing statistical comparison between individual exercise intensities versus the aforementioned double linear model.

#### Surface electromyography

Myoelectric activity was assessed using surface electromyography (EMG) obtained from the *m. vastus lateralis* of the left leg using a single differential wireless EMG electrode (IWorx Systems Inc, Dover, NH). The electrodes were placed on the belly of the muscle as determined by anatomical landmarks, palpitation, and strong electrical activity when the muscle was voluntarily contracted. The EMG data were collected at a sample rate of 1000 Hz and stored for offline analysis. The raw EMG signals were rectified and processed with a band‐pass filter (10–500 Hz). Each electrical burst corresponding to a muscle contraction was detected and the signal amplitude characteristics were analyzed via root mean square (RMS) for the final five contraction cycles (i.e., a 10‐sec window) centered upon each 1‐min interval of the incremental cycling test to provide an index of muscle activation and motor neuron firing rate. Similar to previous investigations (Beck et al. 2005; Rouffet and Hautier 2008; Boone et al. 2012), RMS values were normalized to the average values measured at 50 W in each test.

### Statistical analysis

All statistical analyses were performed using a commercially available software package (SigmaPlot/SigmaStat12.5, Systat Software, Point Richmond, CA). Paired t‐test was used to test for the differences in the FMD response, $\dot{V}O_{2\max}$, and maximum workload before and 48 h after the EIMD procedure. One‐way repeated measures analysis of variance (condition × exercise intensity) with Student–Newman–Keuls post hoc analyses were performed to identify significant changes between pre‐ and 48 h post‐EIMD [HHb], TSI%, and EMG RMS responses. Statistical significance was declared when *P* ≤ 0.05.

## Results

### Muscle damage

Eccentric exercise of the quadriceps resulted in a significant decline in MVC in both quadriceps 48 h post exercise (*P* < 0.05). On average, force declined in the right leg from 644 ± 49 N to 488 ± 53 N (~20% decrease) and in the left leg from 552 ± 47 N to 479 ± 34 (~13% decrease). Additionally, VAS assessed quadriceps muscle soreness increased from 0.15 to 1.83 ± 0.23 cm (*P* < 0.05). These changes are consistent with the development of mild‐to‐moderate EIMD. There was no correlation between decline in MVC and FMD post‐EIMD (*P* < 0.05).

### Endothelial function

**Figure 1:**
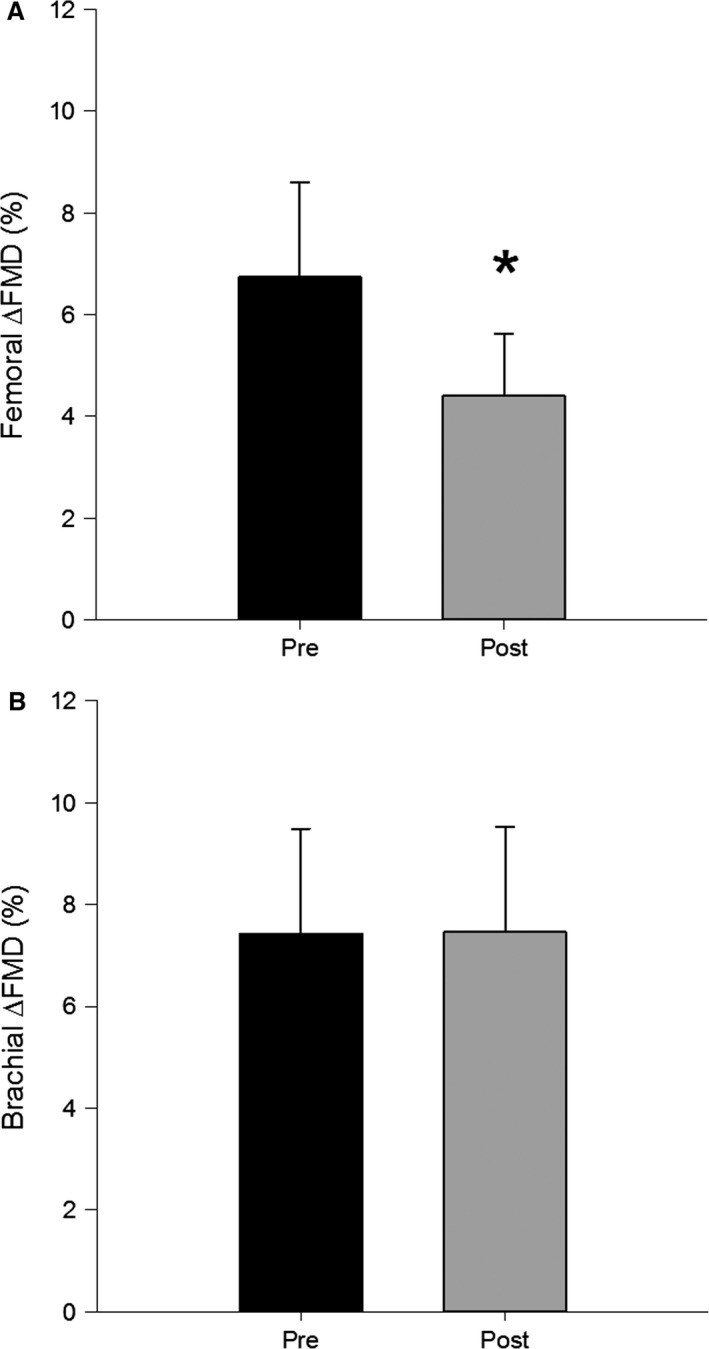
Mean femoral (A) and brachial (B) %Δ flow‐mediated dilation response pre‐ and 48 h post‐EIMD. *denotes significance at *P* < 0.05.

Absolute baseline diameters for both superficial femoral (pre, 5.97 ± 0.21; post, 5.92 ± 0.05 mm) and brachial arteries (pre, 3.83 ± 0.11; post, 3.85 ± 0.14 mm) were not different 48 h post EIMD (*P* > 0.05). Forty‐eight hours post‐EIMD, superficial femoral artery FMDmmΔ significantly decreased from 0.39 ± 0.05 mm to 0.23 ± 0.06 mm. A similar decrease in FMD%Δ was observed 48 h post‐EIMD, such that superficial femoral endothelial function decreased ~ 40% from 6.75 ± 3.89% to 4.01 ± 2.90% following muscle damage (Fig. 1). This decrease in superficial femoral artery FMD remained significant after normalizing for the SS_AUC_ to peak dilatory response (%FMD/SS_AUC_) (Table 1). Brachial artery FMDmmΔ and FMD%Δ were similar pre‐ and post‐EIMD. Postdamage microvascular reactivity measured as the peak postocclusive reactive hyperemic velocities in the brachial (pre, 62.93 ± 3.68 m sec^−1^; post, 65.63 ± 3.04 m sec^−1^; *P* = 0.47) and superficial femoral arteries (pre, 50.69 ± 1.73 m sec^−1^; post, 54.78 ± 2.26 m sec^−1^; *P* = 0.26) were not different 48 h post‐EIMD compared to pre‐EIMD.

**Table 1:** Selected vascular and hemodynamic variables

	Pre	Post
Brachial		
FMD Shear, sec^−1^	24631.9 ± 3277.24	30045.8 ± 3810.25
FMD normalized, mmΔ	1.23E‐06 ± 4.00E‐06	8.71E‐07 ± 3.50E‐06
FMD normalized, %Δ	3.49E‐05 ± 1.38E‐05	2.56E‐05 ± 1.00E‐05
Femoral		
FMD Shear, sec^−1^	12147.8 ± 1851.86	12444.5 ± 940.21
FMD normalized, mmΔ	4.26 E‐06 ± 1.10E‐06	2.15E‐06 ± 0.65E‐06*
FMD normalized, %Δ	6.98E‐05 ± 1.68E‐05	3.62E‐05 ± 1.03E‐05*
Hemodynamic		
Resting mean arterial pressure, mmHg	85.69 ± 2.35	83.23 ± 1.88
Heart rate max, beats per min	186 ± 2.99	183 ± 2.75

Values are means ± SE. *P < 0.05 versus Pre.

### Incremental exercise

**Figure 2:**
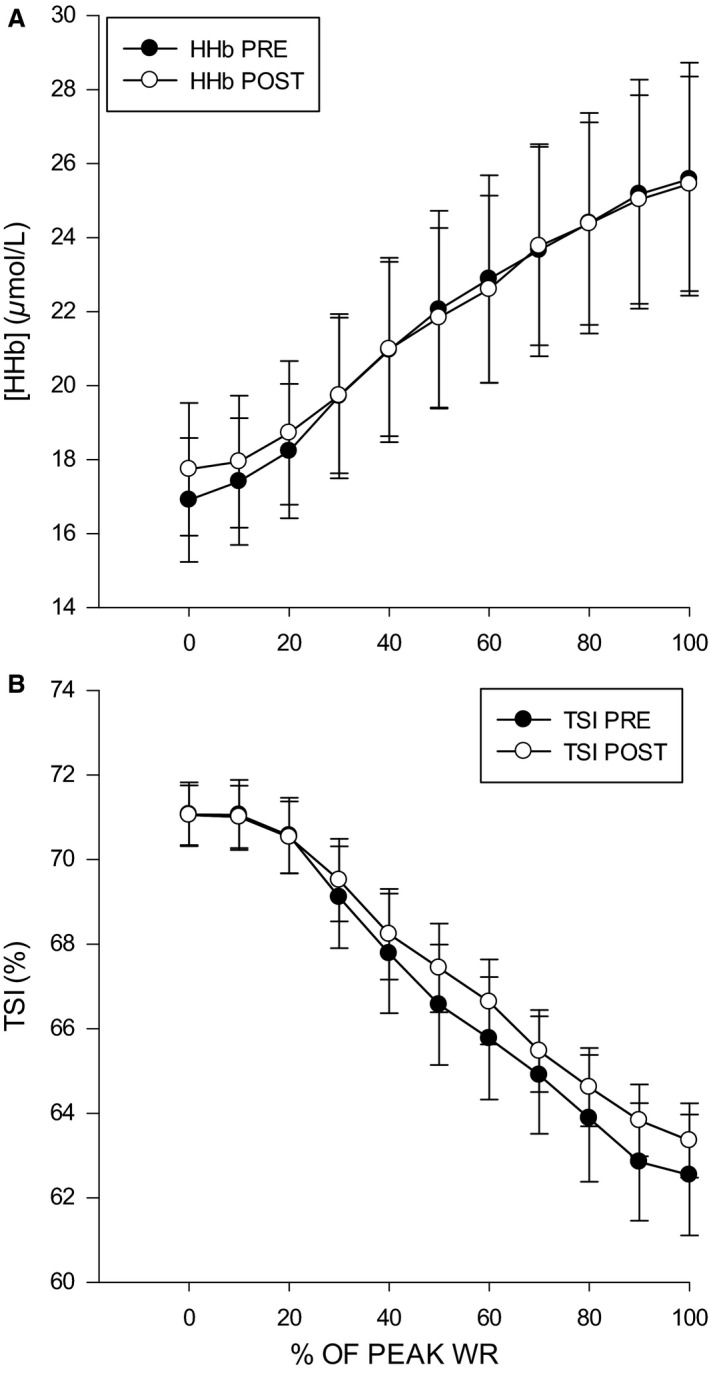
Mean NIRS values of the right *vastus lateralis*. (A) Relative change in deoxyhemoglobin concentration [HHb] μmol L^−1^ pre‐ and 48 h post‐EIMD. (B) Relative change in tissue saturation index (TSI)% pre‐ and 48 h post‐EIMD. *denotes significance at *P* < 0.05.

**Figure 3:**
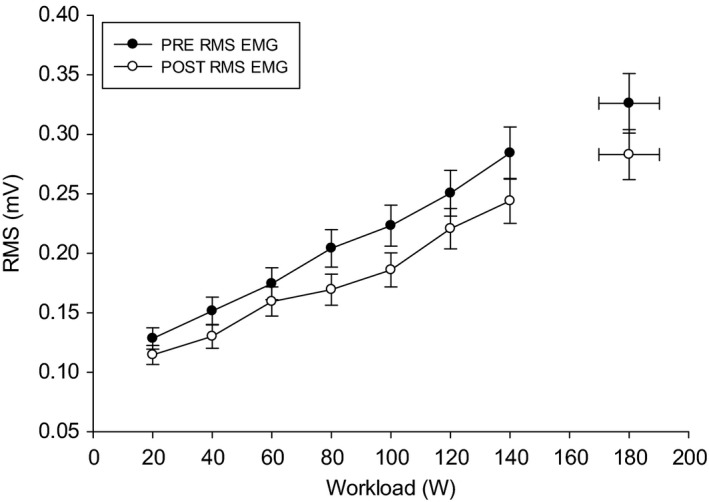
Pre‐ and 48 h post root mean square EMG response of the left *vastus lateralis*. *denotes significance at *P* < 0.05.

Following eccentric muscle damage, the increase in [HHb] above baseline during the exercise was not different compared to pre‐EIMD (pre, 16.91 ± 4.69 μm; post, 17.74 ± 4.9; *P* = 0.17). Similarly, the increase in TSI% was not different pre‐ and post‐EIMD (pre, 71.06 ± 19.7%; post, 71.04 ± 19.7%; *P* = 0.94). At each progressive increase in exercise intensity (i.e., 0–100% peak), the [HHb] and TOI% responses were similar pre‐ and 48 h post‐EIMD (*P* > 0.05) (Fig. 2). Thus, O_2_ extraction during ramp‐incremental exercise was not different following eccentric muscle damage. EMG RMS of the left *m. vastus lateralis* from 20 W to 140 W and peak RMS were not different post‐EIMD compared to pre (*P* > 0.05), (Fig. 3). PPO was similar pre‐ (231 ± 38 W) and 48 h post‐EIMD (229 ± 37 W). Post‐EIMD $\dot{V}O_{2\max}$ (2.96 ± 0.16 L min^−1^) was not significantly different compared to pre (3.01 ± 0.18 L min^−1^; *P* > 0.05).

## Discussion

Eccentric exercise results in mechanical disruption that effects gross skeletal muscle function (Clarkson and Hubal 2002). Beyond the myocyte, muscle damage is associated with increases in central artery stiffness and local endothelial dysfunction (Miyachi et al. 2004; Barnes et al. 2010). However, to the best of our knowledge, no studies have investigated the impact of EIMD on localized and systemic resting macrovascular and microvascular function as well as the dynamic microvascular responses to incremental exercise. The major findings of this study were that 48 h after the eccentric exercise protocol, local endothelial dependent flow‐mediated dilation in the damaged limb (i.e., superficial femoral artery) was significantly decreased. However, our nonlocalized index of systemic endothelial‐dependent dilation flow‐mediated dilation (i.e., brachial artery) was not affected. Within the microcirculation, our measurements of resting microvascular reactivity, as assessed via the postocclusive reactive hyperemic response, were not altered 48 h post damage. These data suggest that following moderate EIMD, the decrease in endothelial function is isolated to the large vessels located in close proximity to the damaged tissue, but this dysfunction does not appear to occur in the microvasculature of the damaged limb. Unique to this study was the evaluation of microvascular function during exercise. Consistent with our resting measurements, EIMD had no impact on the [HHb] or TSI% responses to ramp‐incremental exercise. Similarly, our measurements of $\dot{V}O_{2\max}$ were unchanged following damage. These data suggest that, while local endothelial function was depressed, moderate muscle damage did not decrease peripheral O_2_ extraction, as assessed via [HHb] and TSI% during exercise. Thus, whereas moderate myocyte damage will decrease the muscle(s) force generating capacity, the capacity to maintain adequate dynamic adjustments to O_2_ extraction, and subsequently maximal aerobic exercise capacity, was not impaired following EIMD in this study.

Unaccustomed eccentric exercise is known to decrease isometric strength, in which these quantitative decreases in force serve as a common method of assessing the degree of muscle damage (Warren et al. 1999). The 13–20% decrease in maximal isometric force observed in this study is consistent with other studies investigating the effects of muscle damage on cardiorespiratory function (Davies et al. 2008, 2011a; Black and Dobson 2012; Black et al. 2015), however, not all studies (Barnes et al. 2010). This coupled with a significant increase in muscle pain suggest that the eccentric exercise protocol we used was effective in producing moderate muscular damage, albeit this may not be similar to damage shown in animal models of running downhill to exhaustion (Kano et al. 2005).

Unlike previous investigations, this is the first study to concurrently investigate the effects of EIMD on local and nonlocalized macro‐ and microvascular function. Specifically, the superficial femoral artery experienced an ~40% reduction in FMD following eccentric muscle damage in the quadriceps, which could be due to decreased endothelial or smooth muscle function. This finding is similar to that of Stacy et al. (2013) who elicited muscle damage in the biceps brachii and observed a decrease in brachial artery FMD. Additionally, these authors were able to determine that the reduced FMD following damage was due to both endothelium‐dependent and ‐independent mechanisms. Local decreases to the FMD response shown herein support a decrease in endothelium‐dependent mechanisms. However, endothelium‐independent changes, while not measured in this study, may mediate a portion of this reduction as well (Smith et al. 2000). While eccentric muscle damage increases inflammatory markers within the systemic circulation (Vlachopoulos et al. 2005; Barnes et al. 2010), the inflammatory response within the damaged tissue may be higher than nondamaged nonlocal muscle tissue, especially for moderate muscle damage. For instance, the proinflammatory cytokines, interleukin (IL)‐1*β* and tumor necrosis factor (TNF)‐*α*, are expressed within skeletal muscle for up to 5 days following eccentric exercise damage (Cannon et al. 1989; Fielding et al. 1993). Conversely, IL‐1*β* and TNF‐*α* in the systemic circulation are only slightly increased following eccentric damage (Brenner et al. 1999; Smith et al. 2000; Toft et al. 2002). These differences may be due in part to the inhibitory action of an antiinflammatory response as indicated by increased levels of plasma IL‐1ra, IL‐10, and soluble TNF‐*α* receptors following damage (Smith et al. 2000; Toft et al. 2002; Peake et al. 2005). While not measured in this study, the localized inflammatory response at the site of muscle damage may have contributed to the decrease in superficial femoral artery flow‐mediated dilation observed in this study, whereas the systemic inflammatory response may not have been sufficient to depress the nonlocal flow‐mediated dilation in the brachial artery.

While the effects of EIMD on superficial femoral artery FMD are likely mediated by the increased local inflammatory responses, there is the potential of resistance exercise alone altering the FMD response (Dawson et al. 2013). However, these reductions are only apparent immediately post exercise (i.e., within 30 min) and normalize within 24–48 h (Dawson et al. 2013). The FMD response following exercise is therefore typically biphasic, which is often observed as an initial (1–24 h) decrease followed by normalization within 24–48 h (Dawson et al. 2013). Thus, current evidence suggests that the decreased FMD response shown in this study at 48 h post‐EIMD are likely not an effect of the exercise stimulus itself, but is a consequence of the muscle damage response. The decreased FMD response may be thought of as the “Hormesis” hypothesis, in that the decrease in FMD may, with repeated bouts of eccentric exercise, become capable of adaptive processes that increase functionality of the cells during these stressors (Padilla et al. 2011). However, what remains to be examined is repeated bouts of EIMD and its effect on FMD response at 24 and 48 h (Jasperse and Laughlin 2006; Whyte and Laughlin 2010).

The peak reactive hyperemic response following a period of temporary arterial occlusion has previously been used as a test of microvascular function (Anderson et al. 2011; Flammer et al. 2012). In this study, eccentric muscle damage had no effect on the postocclusive reactive hyperemic response in the leg, despite a decreased superficial femoral artery flow‐mediated dilation. Unlike the flow‐mediated dilation response that is largely, but not solely, nitric oxide dependent (Harris et al. 2010), the peak hyperemic response is mediated primarily by the activation of inwardly rectifying potassium channels with minor contributions from nitric oxide and prostaglandins (Engelke et al. 1996; Crecelius et al. 2013). Therefore, the findings of this study indirectly suggest that moderate muscle damage may not have an adverse impact on the activation of inward rectifying potassium channels in the smaller resistance arterioles that determine the microvascular peak postocclusive hyperemic response.

During ramp‐incremental exercise, the increase in fractional O_2_ extraction, as measured by the [HHb] signal via NIRS, occurs in response to dynamic changes within the microcirculation (Boone et al. 2012, 2016; Murias et al. 2013; McLay et al. 2016). A key finding of this study is the unchanged [HHb] and TSI% response to incremental exercise following eccentric muscle damage. Given that the NIRS signal comes from the small arterioles, venoules, and capillaries, this finding suggests that moderate muscle damage has minimal impact on microvascular function. This finding is at odds with previous work in animal models of EIMD who have clearly demonstrated the potential for microvascular dysfunction both at rest and during exercise (Kano et al. 2004). Similarly, in a human model of eccentric muscle damage, Davies et al. (2008) demonstrated that EIMD slows severe‐intensity [HHb] onset kinetics and the $\dot{Q}O_{2}$:$\dot{V}O_{2}$ ratio, but does not change $\dot{V}O_{2}$ kinetics 48 h following eccentric damage. One possible explanation for these discrepant results could be related to the magnitude of EIMD induced in the studies. It is possible that severe EIMD elicited in this study could shift the $\dot{Q}O_{2}$:$\dot{V}O_{2}$ ratio in an attempt to compensate for the decreases in capillary O_2_ pressure and O_2_ flux to maintain continuous adjustments to $\dot{V}O_{2}$. However, like that seen in animals, the difference between control and damaged conditions was only observed over the first 5–20 sec of exercise and no differences in the [HHb] amplitude or steady state were detected (Davies et al. 2008). It should, however, be highlighted that not all studies report an altered [HHb] kinetics following EIMD. Recently, Nederveen et al. (2014) report no impact at 48 h after EIMD on moderate intensity [HHb] kinetics, however, at 24 h, one group did show speeded kinetics. As such, it is possible the current examination of O_2_ extraction missed important changes at 24 h.

Unique to this study was the use of ramp‐incremental exercise such that it allowed for investigation of the continuous dynamic microvascular [HHb] response as a function of exercise intensity. We observed no difference in the amplitude increase in [HHb] or TOI% from rest to peak exercise. Similarly, there were no differences in these variables at each investigated submaximal workload. Previous kinetic studies strongly suggest that more severe eccentric muscle damage alters the microvascular response at early exercise onset (i.e., first 5–20 sec) (Kano et al. 2005; Davies et al. 2008), but our findings suggest that moderate EIMD has no impact on the ramp‐incremental responses. Interestingly, the decreased superficial femoral FMD had no impact on the NIRS response during exercise. However, the FMD response may only be essential for the initial hyperemic response at exercise onset (i.e., 5–20 sec) (Wray et al. 2005). Thus, the consequences of an impaired superficial femoral artery FMD via EIMD may be short‐lived during exercise.

The extent to which EIMD impacts maximal oxygen uptake remains unclear with previous studies demonstrating reductions (Black and Dobson 2012; Black et al. 2015) in $\dot{V}O_{2\mathrm{peak}}$, during both cycling and running, and studies finding no impact of EIMD on $\dot{V}O_{2\mathrm{peak}}$ (Warren et al. 1999; Davies et al. 2011b). A strength of this study was our use of a validation protocol (i.e., established $\dot{V}O_{2}$ plateau) per published guidelines (Poole et al. 2008) to obtain a more “true” estimate of $\dot{V}O_{2\max}$ rather than using secondary criteria such as attainment of a percentage of max heart, attainment of a critical RER value, and/or RPE to determine if a maximal effort was provided. Therefore, the previously observed decreases in $\dot{V}O_{2\mathrm{peak}}$ could be due to several factors (e.g., pain, kinematics, etc.) in addition to changes in O_2_ delivery and utilization. However, our unchanged validated measurements of $\dot{V}O_{2\max}$ are consistent with the unchanged [HHb] and TSI% responses observed post damage and suggests that mild‐to‐moderate EIMD has little to no impact on convective and diffusive factors that determine $\dot{V}O_{2\max}$.

### Experimental considerations

Several methodological considerations are relevant to the interpretation of the present investigation. Prior to the start of the study, subjects were not familiarized with the maximal cycling procedures, which may cause unwanted changes in plasma fluid following the first maximal exercise bout (Gillen et al. 1991). Secondly, the placement of the NIRS device on the *m. vastus lateralis* prevented investigation of the quadriceps; however, our placement was consistent with previous NIRS and EIMD cycling studies (Ferreira et al. 2007; Davies et al. 2008). The assumptions and limitations relevant to the NIRS technique have been previously been discussed in detail (Kowalchuk et al. 2002; DeLorey et al. 2003; Ferreira et al. 2005) and will only be highlighted here. Briefly, the NIRS‐derived [HHb] is reflective of changes in hemoglobin oxygenation within the small arterioles, venoules, capillaries, and intracellular myoglobin due to similar absorption properties of the NIRS light wavelengths, thus preventing distinction between the two (Davis and Barstow 2013). However, the [HHb] signal has previously been used to evaluate microvascular O_2_ exchange and its kinetic response to exercise is not different from direct microvascular partial pressure of oxygen measurements (Koga et al. 2012). In addition, the NIRS system used in this study cannot evaluate changes in muscle heterogeneity (Okushima et al. 2015) and future studies are needed to more completely evaluate the NIRS responses across different muscles and muscle depths following muscle damage. A third consideration is the potential for changes in motor activation patterns of the anterior and posterior musculature during cycling with EIMD. However, we observed no difference in *m. vastus lateralis* activation suggesting that motor activation patterns were unaltered. We report changes at 48 h, but did not measure vascular function at 24 h. This may have allowed important changes in $\dot{V}O_{2\max}$, NIRS, and FMD to be missed, but it is in line with reports of measurements taken at 48 h (Barnes et al. 2010; Davies et al. 2011a; Black and Dobson 2012).

## Conclusions

In conclusion, unaccustomed eccentric exercise eliciting moderate muscle damage showed a decrease in local superficial femoral artery vascular function, but the index of systemic arterial function was not altered at 48 h. These data suggest that local factors may inhibit macrovascular function post‐EIMD, but only occur relative to the damaged tissue. In addition, the evaluation of dynamic adjustments to peripheral O_2_ extraction and utilization as assessed by [HHb], TOI%, and $\dot{V}O_{2\max}$ were unaltered during ramp‐incremental exercise. These data suggest that microvascular function related to O_2_ extraction is minimally impacted during exercise following moderate muscle damage. Finally, future work may look into comparing different levels of EIMD; this would be beneficial to observe if there was a level of damage needed to observe these physiological dysfunctions in humans previously shown in animals.

## Conflict of Interest

None declared.
